# SARS-CoV-2 Spike Expression at the Surface of Infected Primary Human Airway Epithelial Cells

**DOI:** 10.3390/v14010005

**Published:** 2021-12-21

**Authors:** Shilei Ding, Damien Adam, Guillaume Beaudoin-Bussières, Alexandra Tauzin, Shang Yu Gong, Romain Gasser, Annemarie Laumaea, Sai Priya Anand, Anik Privé, Catherine Bourassa, Halima Medjahed, Jérémie Prévost, Hugues Charest, Jonathan Richard, Emmanuelle Brochiero, Andrés Finzi

**Affiliations:** 1 Centre de Recherche du CHUM (CRCHUM), Montréal, QC H2X 0A9, Canada; shilei.ding@mail.mcgill.ca (S.D.); damien.adam@umontreal.ca (D.A.); guillaume.beaudoin-bussieres@umontreal.ca (G.B.-B.); alexandra_tauzin@hotmail.fr (A.T.); shang.gong@mail.mcgill.ca (S.Y.G.); romain.gasser@umontreal.ca (R.G.); annemarie.laumaea@umontreal.ca (A.L.); sai.anand@mail.mcgill.ca (S.P.A.); anik.prive.chum@ssss.gouv.qc.ca (A.P.); catherine.bourassa.chum@ssss.gouv.qc.ca (C.B.); halima.medjahed.chum@ssss.gouv.qc.ca (H.M.); jeremie.prevost@umontreal.ca (J.P.); jonathan.richard.1@umontreal.ca (J.R.); emmanuelle.brochiero@umontreal.ca (E.B.); 2 Département de Médicine, Université de Montréal, Montréal, QC H2X 0A9, Canada; 3 Département de Microbiologie, Infectiologie et Immunologie, Université de Montréal, Montréal, QC H2X 0A9, Canada; 4 Department of Microbiology and Immunology, McGill University, Montreal, QC H3A 2B4, Canada; 5 Laboratoire de Santé Publique du Québec, Institut Nationale de Santé Publique du Québec, Sainte-Anne-de-Bellevue, QC H9X 3R5, Canada; hugues.charest@inspq.qc.ca

**Keywords:** COVID-19, SARS-CoV-2, spike glycoproteins, nucleocapsid, authentic virus, human primary airway epithelial cells, convalescent plasma, mRNA vaccine, neutralization, antibody-dependent cellular cytotoxicity (ADCC)

## Abstract

Different serological assays were rapidly generated to study humoral responses against the SARS-CoV-2 Spike glycoprotein. Due to the intrinsic difficulty of working with SARS-CoV-2 authentic virus, most serological assays use recombinant forms of the Spike glycoprotein or its receptor binding domain (RBD). Cell-based assays expressing different forms of the Spike, as well as pseudoviral assays, are also widely used. To evaluate whether these assays recapitulate findings generated when the Spike is expressed in its physiological context (at the surface of the infected primary cells), we developed an intracellular staining against the SARS-CoV-2 nucleocapsid (N) to distinguish infected from uninfected cells. Human airway epithelial cells (pAECs) were infected with authentic SARS-CoV-2 D614G or Alpha variants. We observed robust cell-surface expression of the SARS-CoV-2 Spike at the surface of the infected pAECs using the conformational-independent anti-S2 CV3-25 antibody. The infected cells were also readily recognized by plasma from convalescent and vaccinated individuals and correlated with several serological assays. This suggests that the antigenicity of the Spike present at the surface of the infected primary cells is maintained in serological assays involving expression of the native full-length Spike.

## 1. Introduction

Very shortly after SARS-CoV-2 was declared a pandemic by the World Health Organization [1], major efforts to understand humoral responses against this new virus were undertaken. Indeed, a myriad of different assays were deployed around the globe, including soluble recombinant forms of the Spike, its receptor-binding domain (RBD), cell-based assays expressing different forms of the Spike, pseudoviral assays, and assays using authentic SARS-CoV-2 and infected cells [2,3,4,5,6,7,8,9,10]. These assays were then used to study vaccine-elicited humoral responses [11,12,13] and to compare these with those elicited by natural infection [11,12,14]. As well as identifying antibody-mediated neutralization as a likely correlate of protection [15,16,17], emerging evidence points to the potential benefits of antibody-mediated effector functions [18,19,20,21]. Some of these Fc-mediated effector functions, such as antibody-dependent cellular cytotoxicity (ADCC) or antibody-dependent cellular phagocytosis (ADCP), require recognition of the antigen at the surface of the infected cells. This raises an important question about expression levels of SARS-CoV-2 Spike at the surface of primary human airway epithelial cells (pAECs) infected with authentic virus.

To address this question, we developed a FACS-based assay combining the cell-surface detection of the Spike and the intracellular detection of the nucleocapsid (N) in pAECs infected with authentic SARS-CoV-2. This assay allows for the distinction between infected (N+) and uninfected (N−) cells. We found that the SARS-CoV-2 Spike is abundantly expressed at the surface of infected pAECs. Spike recognition at the surface of the infected pAECs strongly correlated with its detection by commonly used serological assays, which include detection at the surface of transfected or transduced cells, pseudoviral neutralization, and Fc-mediated effector functions such as ADCC. Measurements were taken with plasma from a cohort of individuals who were SARS-CoV-2 naïve and vaccinated and of those who were previously infected.

## 2. Materials and Methods

### 2.1. Ethics Statement

All subjects gave their informed consent for inclusion before they participated in the study. The study was conducted in accordance with the Declaration of Helsinki. Primary human airway epithelial cells (pAECs) isolated from the lung biopsies of healthy individuals were provided by the CRCHUM’s Respiratory Cell and Tissue Biobank from the Respiratory Health Research Network of Québec with informed written consent prior to enrolment (protocol #08.063) and approval of the research study (protocol #20.454, approved on 6 April 2021) by the CRCHUM Institutional Review Board. Convalescent plasma and plasma from vaccinated individuals were obtained from donors who consented to participating in this research project at CHUM (protocol #19.381, approved on 25 March 2020). The convalescent plasma donors met all donor eligibility criteria: previously confirmed COVID-19 infection and the complete resolution of symptoms for at least 14 days.

### 2.2. Plasmids

The plasmid expressing the SARS-CoV-2 Spike glycoprotein was kindly provided by Stefan Pöhlmann (Georg-August University, Göttingen, Germany) and has been previously reported [22]. The pNL4.3 R-E-Luc was obtained from the NIH AIDS Reagent Program.

### 2.3. Primary Cells and Viruses

Primary human airway epithelial cells (pAECs) were isolated from bronchial biopsies collected from two healthy subjects (males with a mean age of 56 years). After recovery, the bronchial tissues were rinsed and then incubated overnight at 4 °C with MEM medium (Life Technologies, Carlsbad, CA, USA) supplemented with 7.5% NaHCO_3_ (Sigma-Aldrich, St. Louis, MO, USA), 2 mM L-glutamine, 10 mM HEPES (ThermoFisher Scientific, Waltham, MA, USA), 0.05 mg/mL gentamycin, 50 U/mL penicillin/streptomycin, 0.25 μg/mL Fungizone (Life Technologies), and 0.1% protease (from Streptomyces griseus; Sigma-Aldrich) and 10 μg/mL DNAse (Deoxyribonuclease I from bovine pancreas; Sigma-Aldrich). The protease and DNAse activities were then neutralized with FBS (Life Technologies). The freshly isolated cells were gently scraped off the remaining tissue and the red blood cells were removed by treatment with ACK lysis buffer (0.1 mM NH_4_Cl, 10 μM KHCO_3_, and 10 nM EDTA). After counting, the freshly isolated pAECs were seeded into flasks coated with Purecol (Cedarlane, Burlington, ON, Canada) and collagen IV (Sigma-Aldrich) in a mix (50:50) of PneumacultEx (STEMCELL Technologies, Vancouver, BC, Canada) and CnT-17 (CellnTec Advanced Cell Systems, Bern, Switzerland) media for two days and then grown in CnT-17 until confluence was reached. The pAECs were then detached with a trypsin solution before being seeded into 100 mm dishes coated with Purecol and collagen IV and being cultured in CnT-17 until confluency (~5–7 days) and then in a mix of DMEM and BEGM (Lonza, Basel, Switzerland) for 2 days before experimentation took place. Authentic SARS-CoV-2 viruses were isolated, sequenced, and amplified from clinical samples obtained from patients infected with SARS-CoV-2 D614G or B.1.1.7 (Alpha variant) by the Laboratoire de Santé Publique du Québec (LSPQ). The virus was sequenced by MinION technology (Oxford Nanopore technologies, Oxford, UK). All work with the infectious SARS-CoV-2 authentic virus was performed in Biosafety Level 3 (BSL3) facilities at CRCHUM using appropriate positive-pressure air respirators and personal protective equipment.

### 2.4. Flow Cytometry Analysis of Cell-Surface Staining

SARS-CoV-2 authentic viruses (D614G or B.1.1.7 (α) variant) were used to infect the pAECs at a multiplicity of infection (MOI) of 0.1. Forty-eight hours after infection, cells were detached by PBS-EDTA (10 mM) and 0.2 × 10^6^ cells per sample were stained with CV3-25 (5 µg/mL) or plasma (1/1000) for 30 min at 37 °C. Alexa Fluor-647-conjugated goat anti-human IgG (H + L) Ab (1/1000, Invitrogen, Waltham, MA, USA.) was used as a secondary antibody to stain the cells for 30 min at room temperature. The cells were then fixed with PBS containing 4% paraformaldehyde for 48 h at 4 °C. Then, the cells were stained intracellularly for SARS-CoV-2 nucleocapsid (N) antigen, using the Cytofix/Cytoperm fixation/permeabilization kit (BD Biosciences) and 1 µg/mL anti-N mAb (clone mBG17; Kerafast, Boston, MA, USA) conjugated with the Alexa Fluor 488 dye according to the manufacturer’s instructions (Invitrogen). The percentage of infected cells (N+ cells) was determined by gating the living cell population based on the viability dye staining (Aqua Vivid, Invitrogen). Samples were acquired on a LSR II cytometer (BD Biosciences, Franklin Lakes, NJ, USA), and data analysis was performed using FlowJo v10.5.3 (BD Biosciences).

### 2.5. Statistical Analyses

Statistics were analyzed using GraphPad Prism version 8.0 (GraphPad, San Diego, CA, USA). Every dataset was tested for statistical normality, and this information was used to apply the appropriate (parametric or nonparametric) statistical test. *p* values < 0.05 were considered significant; significance values are indicated as * *p* < 0.05, ** *p* < 0.01, *** *p* < 0.001, and **** *p* < 0.0001.

## 3. Results and Discussion

### 3.1. Spike Recognition at the Surface of Infected Primary Human Airway Epithelial Cells

**Figure 1:**
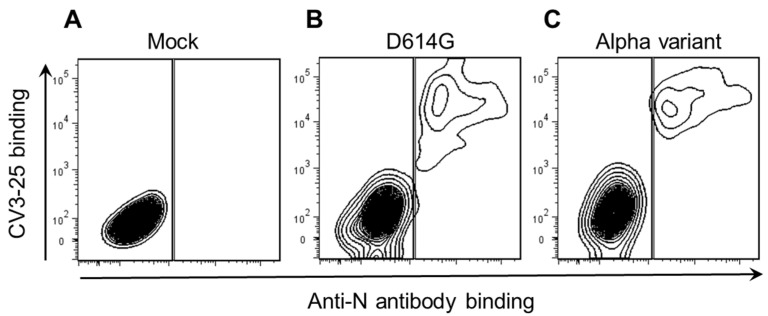
Concomitant detection of the SARS-CoV-2 surface Spike and intracellular nucleocapsid antigens in pAECs. pAECs (cell preparations from two different subjects) were either (**A**) mock-infected or (**B**) infected with authentic SARS-CoV-2 D614G or (**C**) infected with Alpha variant of concern. Cells were stained intracellularly with Alexa Fluor 488 pre-coupled anti-nucleocapsid (anti-N) Ab (clone mBG17) for the identification of infected cells. (**A**–**C**) Flow cytometry contour plots showing representative staining with the SARS-CoV-2 anti-S2 CV3-25 antibody.

To measure cell surface expression of the SARS-CoV-2 Spike, we infected pAECs with the SARS-CoV-2 authentic virus. Pre-coupled anti-nucleocapsid (anti-N) antibodies were used to distinguish infected from uninfected bystander cells (Figure 1). Using the conformational independent SARS-CoV-2 S2-specific CV3-25 antibody [18,23,24,25,26], we detected high levels of the Spike at the surface of the infected N+ cells. CV3-25 specifically bound to infected (N+) pAECs when the cells were infected with the authentic D614G (Figure 1B) or Alpha (B.1.1.7) variant of concern (Figure 1C).

### 3.2. Recognition of Infected Human Primary Airway Epithelial Cells by Plasma from Individuals Who Were Previously Infected, or SARS-CoV-2 Naïve and Vaccinated 

**Figure 2:**
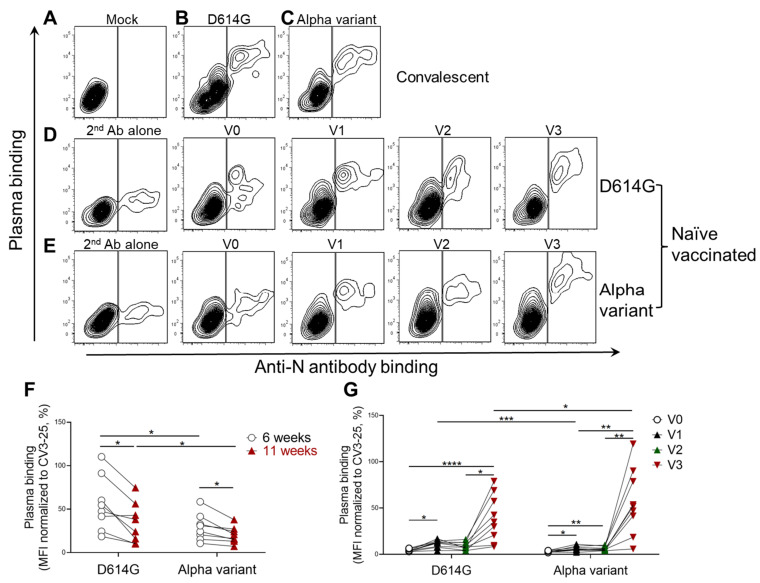
Plasma from individuals infected by SARS-CoV-2 or BNT162b2 vaccinated donors recognized Spike at the surface of human pAECs infected with authentic viruses. pAECs (from two different subjects) were either (**A**) mock-infected or (**B**,**D**,**F**,**G**) infected with authentic SARS-CoV-2 D614G or (**C**,**E**,**F**,**G**) the Alpha variants. In (**A**–**C**,**F**), plasma recovered from eight convalescent individuals whose symptoms had begun 6 or 11 weeks previously was used to stain infected pAECs. Data shown consist of a representative staining of infected pAECs with the plasma from one donor (**A**–**C**), and the median fluorescence intensities (MFI) obtained on N+ cells with plasma from all convalescent donors normalized to the CV3-25 antibody (**F**). In (**D**,**E**,**G**), plasma from nine individuals who were SARS-CoV-2 naïve and vaccinated obtained before vaccination (V0–open circle), 3 weeks (V1–black triangle) and 12 weeks (V2–green triangle) after first mRNA dose, or 3 weeks after the second dose (V3–red triangle) (administered with a 16-week interval between doses) were used to detect infected pAECs. Data shown consist of a representative staining of pAECs infected with SARS-CoV-2 D614G (**D**) or Alpha variant (**E**) with plasma from one donor, and the MFI obtained on N+ cells with plasma from all vaccinated individuals normalized to the CV3-25 antibody (**G**). Statistical significance was tested using RM one-way ANOVA (**F**, Alpha variant in **G**), Frieman test (D614G in **G**), and paired *t* test (between D614G and Alpha variant in **F** and **G**) (* *p* < 0.05; ** *p* < 0.01, *** *p* < 0.001, and **** *p* < 0.0001).

We then measured the recognition of the infected pAECs with plasma from eight convalescent individuals whose symptoms had begun 6 and 11 weeks previously (PSO) (Figure 2A–C,F) [5] and plasma from nine individuals who were SARS-CoV-2 naïve, obtained before vaccination (V0), at 3 weeks (V1) and 12 weeks (V2) after the first dose of the BNT162b2 mRNA vaccine, or at 3 weeks after the second dose (V3) (Figure 2D,E,G). The second dose was administered with an interval of 16 weeks between doses [27]. Representative flow cytometry contour plots of the specific recognition of (N+) pAECs infected with SARS-CoV-2 D614G (Figure 2B,D) or the Alpha variant (Figure 2C,E) are shown. Results obtained with all tested plasmas are summarized in Figure 2F,G. It was found that plasma from convalescent or vaccinated individuals specifically bound to infected (N+) pAECs (Figure 2A–E). In line with previous findings that showed a decline in anti-SARS-CoV-2 Spike-specific antibodies after natural infection [3,4,9,28,29], we observed a significant reduction in the recognition of infected pAECs by plasma recovered 11 weeks PSO compared with plasma recovered from the same individuals 6 weeks PSO (Figure 2F). This was observed for pAECs infected with both authentic D614G and Alpha variants. It should be noted that the convalescent plasma used in this study was collected during the first wave of COVID-19 in the province of Quebec, Canada, which was caused by the original Wuhan strain.

When evaluating the recognition of infected pAECs with plasma from individuals who are SARS-CoV-2 naïve, we observed weak but specific recognition using plasma collected before vaccination (V0) (Figure 2D,E,G). This is likely due to the presence of cross-reactive antibodies against other human coronaviruses recognizing the highly conserved S2 subunit [4,30,31,32]. Vaccination of these individuals elicited antibodies that readily recognized pAECs infected with the D614G (Figure 2D,G) or the Alpha variant (Figure 2E,G).

### 3.3. Recognition of SARS-CoV-2 Infected pAECs Correlates with Spike Recognition at the Surface of 293T Cells, Pseudoviral Neutralization, and ADCC

**Figure 3:**
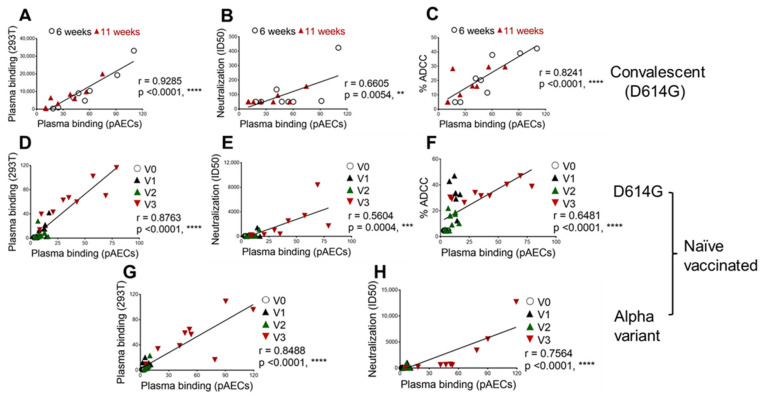
Recognition of SARS-CoV-2 infected cells correlates with recognition of 293T cells expressing Spike, pseudoviral neutralization and ADCC. Recognition of SARS-CoV-2 D614G (**A**–**F**) or Alpha (**G**,**H**) variants infected (N+) pAECs by plasma from convalescent (**A**–**C**) or vaccinated individuals (**D**–**H**) correlates with (**A**,**D**,**G**) binding to 293T Spike expressing cells [5,27], (**B**,**E**,**H**) pseudovirus neutralization [5,27], and (**C**,**F**) ADCC activity using CEM-NKr cells stably expressing SARS-CoV-2 Spike [5,27]. Panels (**A**–**C**) show results generated using plasma from convalescent donors recovered 6 (open circle) or 11 (red triangle) weeks after the onset of symptoms. Panels (**D**–**H**) show data generated using plasma from nine individuals who were SARS-CoV-2 naïve and vaccinated obtained before vaccination (V0—open circle), at 3 weeks (V1—black triangle) and 12 weeks (V2—green triangle) after the first mRNA dose, or 3 weeks after the second dose (V3—red triangle) (administered with a 16-week interval between doses). Statistical significance was tested using Pearson (**A**–**C**) or Spearman (**D**–**H**) rank correlation tests based on statistical normality (** *p* < 0.01, *** *p* < 0.001, and **** *p* < 0.0001).

We then evaluated whether recognition of the Spike at the surface of the infected pAECs correlated with results generated via the serological assays normally used to study humoral responses in individuals who were infected/convalescent or vaccinated [5,27]. We observed that plasma recognition at the surface of infected (N+) cells significantly correlated with the binding of 293T cells expressing the full-length SARS-CoV-2 Spike (Figure 3A,D,G). Similarly, we observed significant correlations with neutralization potency using a well-established pseudoviral assay (Figure 3B,E,H) [4,5,11,18,23,24,33]. Since emerging evidence points to the potential benefits of antibody-mediated effector functions [18,19,20], we also evaluated whether ADCC correlated with recognition of the Spike at the surface of the infected cells. The ADCC assay was performed with the CEM-NKr cell line stably expressing the SARS-CoV-2 Spike as target cells and the PBMCs from healthy donors as effector cells. This assay has been described in detail elsewhere [5,11,21,34]. Interestingly, we observed that recognition of the Spike at the surface of the infected pAECs correlated significantly (*p* < 0.0001) with ADCC (Figure 3C,F).

A limitation of our study is the relatively low number of individuals analyzed; however, we note that our results that used authentic SARS-CoV-2-infected pAECs are consistent with the findings of many widely used serological assays [5,27]. Overall, our results show that the SARS-CoV-2 Spike is abundantly expressed at the surface of infected human pAECs. This confirms that SARS-CoV-2-infected cells represent a potential target for Fc effector responses. Moreover, it suggests that the antigenicity of Spike present at the surface of infected pAECs, following the natural route of Spike cellular trafficking, samples a conformation that is maintained in widely used serological assays involving expression of the native full-length Spike.

## Data Availability

Not applicable.
